# The use of dual mobility cups in revision total hip arthroplasty for failed large head metal-on-metal bearings

**DOI:** 10.1007/s00264-023-06017-z

**Published:** 2023-11-01

**Authors:** Samir Meriem, Alexander Antoniadis, Michele Palazzuolo, Julien Wegrzyn

**Affiliations:** Department of Orthopedic Surgery, Lausanne University Hospital and University of Lausanne, Avenue Pierre-Decker, 4, CH-1011 Lausanne, Switzerland

**Keywords:** Total hip arthroplasty (THA), Adverse reaction to metal debris (ARMD), Dual mobility cup (DMC)

## Abstract

**Purpose:**

Revision of failed large head metal-on-metal (MoM) total hip arthroplasty (THA) is a challenging procedure particularly to reconstruct acetabular bone defect due to osteolysis and to achieve hip stability due to soft tissue damages, both potentially caused by adverse reaction to metal debris (ARMD). This study aimed to evaluate the outcome of dual mobility cup (DMC) constructs in revision THA for failed large head MoM bearings with a special attention to the occurrence of dislocation or re-revision.

**Methods:**

Between 2015 and 2019, 57 patients (64 THAs, 41 men, mean age = 65 ± 10 years) underwent revision for MoM THA with the use of DMC were prospectively included in our total joint registry. Mean time to revision was 11 ± 2.5 years. The causes for revision were adverse reaction to metal debris (ARMD) in 49 THAs (76%), painful hip with elevated blood cobalt-chromium ions in seven (11%), and acetabular aseptic loosening in eight (13%). The revision was complete in 22 THAs (34%) and acetabular only in 42 (66%). Clinical and radiographic outcomes, complications, and re-revisions were evaluated at most recent follow-up.

**Results:**

At mean follow-up of six ± 1.5 years, the pre- to postoperative Harris Hip Score improved from 74 ± 19 to 92 ± 4 (*p* = 0.004). Complications occurred in 11 cases (17%): five dislocations (8%), three periprosthetic infections (5%), two aseptic loosening of the acetabular component (3%), and two periprosthetic fractures (3%). Re-revision was required in six cases (9%).

**Conclusion:**

The use of DMC is a reliable option to prevent instability and ensure a stable acetabular reconstruction in revision THA for failed large head MoM bearings. However, dislocation after revision remains a concern, particularly in cases of severe soft tissue damage related to ARMD.

## Introduction

Large head metal-on-metal (MoM) total hip arthroplasty (THA) was developed as an alternative to conventional metal- or ceramic-on-polyethylene bearings in an attempt to decrease the risk of wear-related osteolysis and aseptic loosening and to prevent instability by increasing the jump distance to dislocation with the use of a large head > 36 mm, both potentially contributing to increase THA survivorship [1]. As many as 1 million large head MoM THA have been implanted in the USA [1, 2]. However, multiple national registries worldwide as well as clinical series have demonstrated higher-than-expected rates of complication with MoM THA at mid- to long-term follow-up [1, 3]. To date, outcomes of MoM THA are known to be poorer than for conventional bearings, with revision rates as high as 15.5% after ten years. Moreover, in the context of adverse reaction to metal debris (ARMD), failed large head MoM THA is frequently associated with severe osteolysis and bone loss, as well as with damaged and compromised soft tissues [4, 5]. In such cases, the surgeon is consequently confronted with technical challenges during revision THA, which could increase the risk of overall complications [6, 7]. Particularly, in the presence of ARMD, severe soft tissue damages could result in hip abductor mechanism deficiency and subsequent dislocation which remains a major concern [8].

The effectiveness of dual mobility cups (DMCs) as implant of choice during acetabular reconstruction to prevent dislocation after revision THA has been extensively reported in the literature, including in the context of severe acetabular bone defect reconstruction and/or hip abductor mechanism deficiency [9, 10]. However, to our knowledge, clinical series specifically dedicated to evaluate the outcomes of revision THA performed with the use of DMC constructs for failed large head MoM bearings remain spare [9, 11]. Therefore, this study aimed to evaluate the occurrence of dislocation or re-revision and analyzed the outcomes of DMC constructs in revision THA for failed large head MoM bearings.

## Materials and methods

### Patients and surgical procedures

**Fig. 1:**
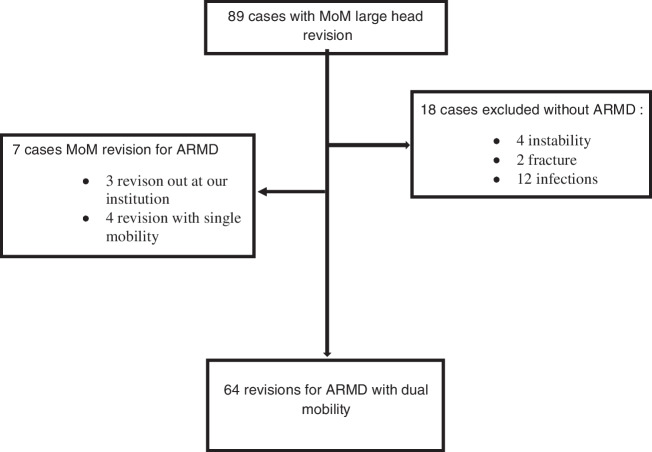
Study design and flowchart

Between 2015 and 2019, 89 revisions of large MoM constructs were identified in our local total joint registry in our hospital, which is an academic centre and a tertiary referral centre. Eighteen revisions for no ARMD-related complications as periprosthetic infection (12 hips), periprosthetic fracture (4 hips), and two aseptic loosening were excluded. Seven cases of ARMD revision; four out at our hospital or three by using a single mobility construct were excluded from this study because of inadequate follow-up or revision with a single mobility construct. Sixty-four revisions for failed MoM THA related to ARMD, painful hip with pathological elevation of blood cobalt-chromium (Co-Cr) ions, or implant aseptic loosening were identified and included in this study to be analyzed at latest follow-up (Fig. 1). The MoM implant revised was Durom® cup construct with Metasul® large Diameter Head and CLS/Sportono® stem (Zimmer, Warsaw, IN, USA). Fifty-seven patients were included (41 men [74%] and 16 women [26%]) with a mean age at revision of 65 ± ten years and a mean BMI of 28 ± 6 kg/m^2^. Mean time to revision was 11 ± 2.5 years (range 4–14 years) (Table 1). The causes for revision were ARMD in 49 THA (76%), painful hip with pathological elevation of blood Co-Cr ions in 7 THA (11%), and acetabular aseptic loosening in eight THA (13%). ARMD was diagnosed using metal artifact reduction sequence magnetic resonance imaging (MARS-MRI), which was performed for all patients with ion values above 119 nmol/l for Co and 135 nmol/l for Cr regardless of the hip pain [12]. In accordance at the classification of lesion based on MRI described by Hauptfleisch et al. [13], 22 patients present a type I lesion (44%), 21 type II (42%), and six type III (12%). In accordance with the recommendations of Swiss Orthopaedics [14], patients with painful hip associated with blood ion levels above 340 nmol/l for Co and 386 nmol/l for Cr underwent revision even in the absence of ARMD on the MARS-MRI evaluation. Acetabular aseptic loosening was evaluated on serial anteroposterior and lateral radiographs of the pelvis and hip by the occurrence of progressive circumferential radiolucent lines and/or evidence of cup migration, according to the criteria of Massin et al. [15]. The mean follow-up was six ± 1.5 years (range 1.5–8 years), with no patients lost to follow-up. Patient informed consent and Institutional Review Board approval were obtained before study initiation (CER-VD #2019–02172).

**Table 1:** Demographic data

Preoperative demographic date	Number	Range	Standard deviation
Female/male	16/41		
Median age (years)	65.7	36–69	10.9
BMI median	28.4	18–42	6.3
Time to revision (years)	11	4–16	2.5
Mean hospital stay (days)	9	2–56	8
Median follow-up (years)	5	0.3–7	1.5

All revision THAs were performed through a conventional posterolateral approach by or under the direct supervision of a senior fellowship-trained hip arthroplasty surgeon at our institution. The revision concerned acetabular and femoral component (complete) in 22 THA (34%) and was acetabular-only in 42 THA (66%). Acetabular and femoral bone defects were graded according to the Paprosky classifications [16]. Soft tissue damages due to ARMD as well as potential hip abductor deficiency were systematically reported on the operative reports. A single design of true hemispherical DMC was used (Symbol®, Dedienne Santé, Mauguio, France) with a cementless press-fit fixation in 53 THA (82%) and a cemented fixation in 11 THA (18%) being cemented in the bon acetabulum in two THA (3%), in a Ganz reinforcement device in three THA(5%), in a Burch-Schneider anti-protrusion cage in one THA (2%), in a porous tantalum reconstruction shell in three THA (5%) or using a double-socket technique (cementation of dual mobility cup in well positioned existing acetabular cup) in two THA (3%) (Table 2).The cementless Symbol® DMC consists of a monoblock Co-Cr metal shell that is double coated with porous titanium alloy plasma spray and hydroxyapatite at the osseointegration surface without additional screwed fixation [17]. The cemented version of the Symbol® DMC was specifically designed for cementation with peripheral radial and concentric circumferential grooves for cement interdigitation [18]. In both component revisions, a cementless modular tapered fluted stem was systematically used for femoral reconstruction with a Restoration Cone-Conical® stem (Stryker, Mahwah, NJ) in seven cases (33%) and a Revision Stem® (Lima Corporate, Udine, Italy) in 14 (66%; Table 2).

**Table 2:** Perioperative data

Perioperative data	Number	Percent	Median Cr-Co ion
Revision indication			
• ARMD	49	76	Cr: 325 ± 200 nmol/l Co: 385 ± 250 nmol/l
• Painful hip with elevated ion•	7	11	Cr: 249 ± 86 nmol/l Co: 243 ± 140 nmol/l
• Loosening	8	12	Cr: 57 ± 25 nmol/l Co: 77 ± 40 nmol/l
Median blood loss (ml)	**Median** 600	**Range** 160–2200	**Standard deviation** 507
Hauptfleisch MRI classification • Type I: Cyst wall < 3 mm • Type II: Cyst wall > 3 mm • Type III: Pseudotumor	**Number** 22 21 6	**Percent** 44 42 12	
Paprosky classification [15]	**Number**	**Percent**	
• Femoral			
- I - II - IIIa	48 14 2	75 22 3	
• Acetabular			
- I - Iia - IIb - IIc - IIIa - IIIb	27 25 4 3 4 1	42 39 6 5 6 2	
Acetabular cup construct	**Number**	**Percent**	
• Cementless • Cemented cup - Ganz - Burch-Schneider - Ultraporous metal back (TMARS) - Double socket - Into bony acetabulum	53 3 1 3 2 2	82 5 2 5 3 3	

### Evaluation

Operative and in-hospital reports were analyzed. Intra-operative blood loss was calculated from fluid accumulation in the suction bottle after subtracting the irrigation fluid and by weighing blood absorbed by surgical gauze. Intraoperative complications were systematically recorded and analyzed.

Patients returned for postoperative follow-up visits at three months, six months, one year, and annually thereafter, where they underwent a physical examination, with clinical outcomes evaluated using the Harris Hip Score (HHS) [19]. Plain anteroposterior and lateral radiographs of the pelvis and affected hip were obtained and analyzed to evaluate implant fixation using the same criteria as described above [15, 16]. Postoperative complications, dislocation reoperation, and re-revision were collected through retrospective chart review.

### Statistical analysis

Quantitative variables are presented as mean ± standard deviation. The comparison of continuous and quantitative variables between the two groups was performed using two-sample *t*-tests, and the comparison of qualitative variables between the two groups was performed using Fisher’s exact tests. Statistical analyses were performed using the SPSS version 22 software (SPSS Inc, Chicago, IL) with a level of significance set at *p* < 0.05.

## Results

### Outcome, complications, and re-revisions

The average follow-up was six ± 1.5 years (range: 4–14 years). The mean preoperative HHS was 74 ± 19 (*r*: 22.6–92.6). Postoperatively, HHS improved for all patients during follow-up and was 76.5 ± 16.5(range: 40–88.9) at three months, 87.8 ± nine (range: 41–92.6) at six months, and 92 ± nine (range: 63.5–95.8) (*p* = 0.004) at the last follow-up.

**Fig. 2:**
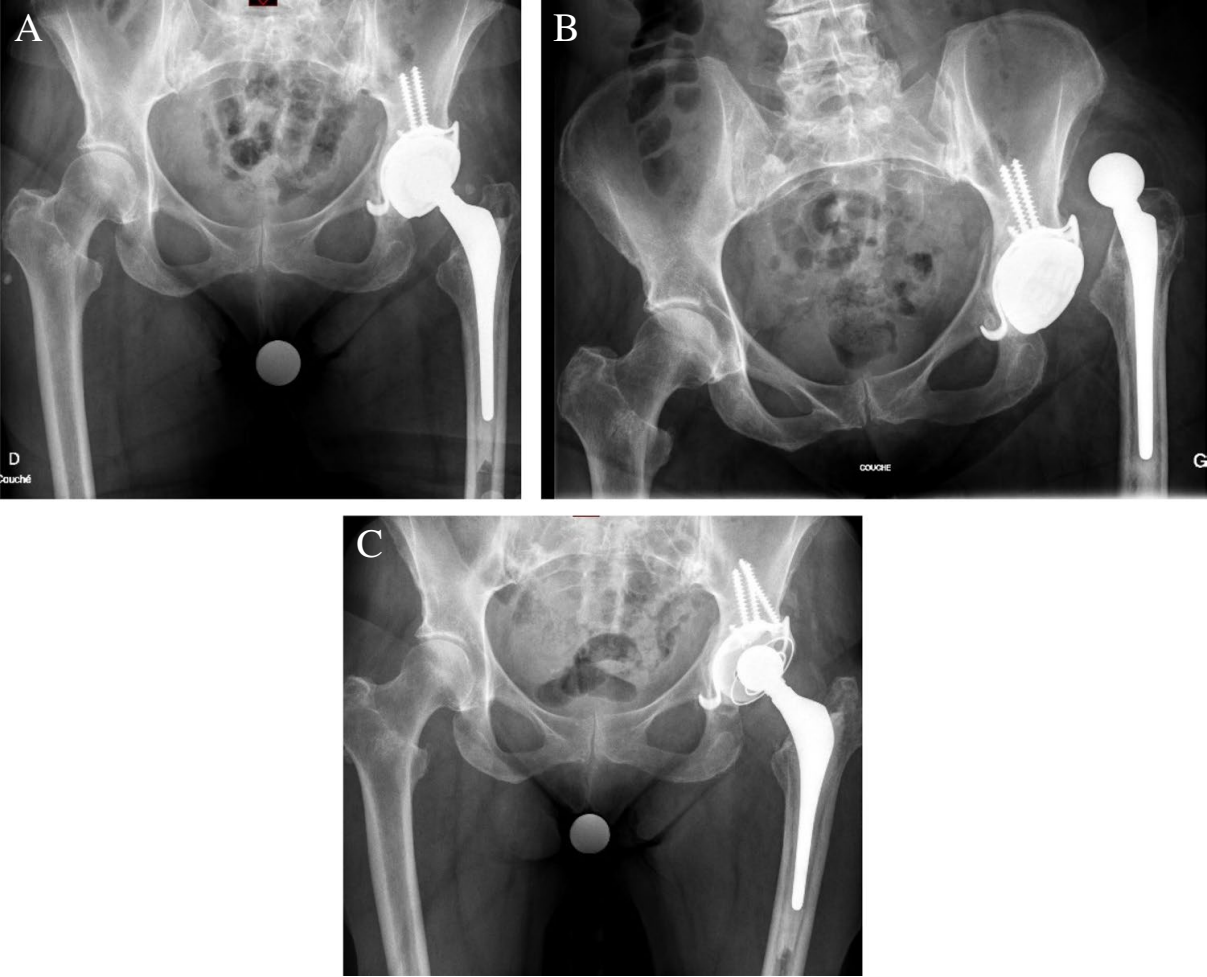
Case of a 72-year-old female patient presenting recurrent instability and required revision to a constrained liner. **A** Postoperative antero-posterior radiograph of the pelvic with revision using cemented dual mobility. **B** At 2 weeks, first dislocation episode. **C** After re-revision using a cemented constrained liner for persistent instability

Early postoperative complications occurred after 11 revision THA (17%), three hips in complete revision group and eight with acetabular revision and only six (9%) required re-revision; two in complete group and four in acetabular group. Dislocation occurred after five revision THA (8%). Three of the dislocation events (6.2%) were a unique occurring at four ± one weeks (SD:1.4) after surgery and managed by closed reduction without recurrence at latest follow-up. All these cases occurred in bipolar revision. However, recurrent dislocation occurred in two THA (acetabular revision only cases) and required re-revision with re-orientation of a cementless DMC that was initially mal-positioned in retroversion in one revision THA, and with conversion to a constrained acetabular component Lefèvre (Lépine Groupe, Genay, France) cemented in a Ganz reinforcement device in one revision THA for the reason of severe hip abductor deficiency due to previous ARMD debridement (Fig. 2). Early periprosthetic joint infection occurred after three revision THA (5%) at seven ± five weeks. Two of these three infections required re-revision with a two-stage procedure (1 of each groups). The last one (acetabular revision) was managed with re-operation for DAIR (debridement, antibiotics, and implant retention).

**Fig. 3:**
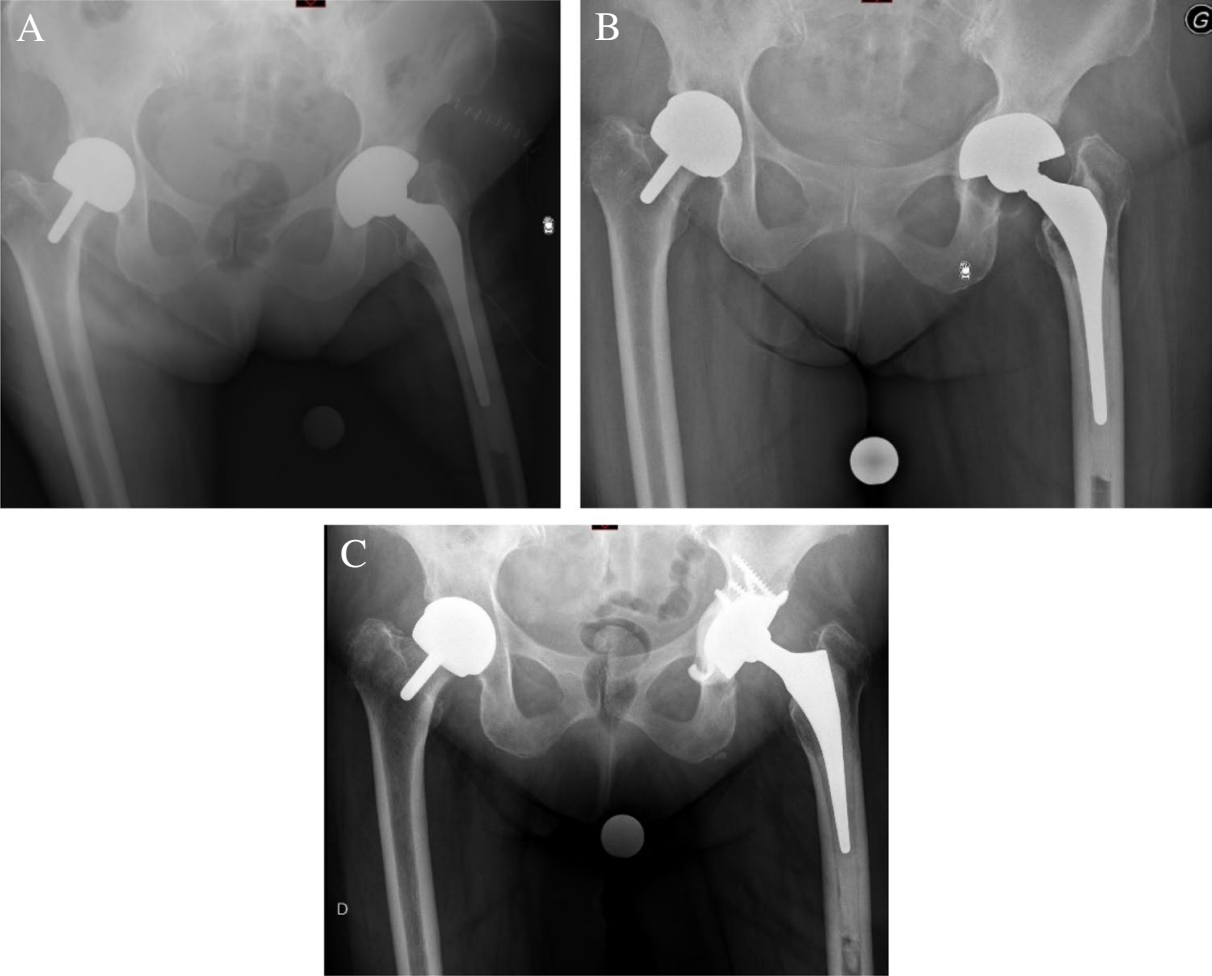
Case of a 92-year-old male patient presenting cup migration, which required revision surgery. **A** Postoperative antero-posterior radiograph of the pelvic with revision using a cementless dual mobility cup. **B** At 6-week follow-up, pelvic radiograph showed aseptic migration of the dual mobility cup. **C** After re-revision using a Ganz reinforcement ring with a cemented dual mobility

Early aseptic loosening occurred after two revision THA (one of each group) (3%) with acetabular reconstruction performed with a cementless DMC and required re-revision at five ± one weeks: one with a cup-cage construct (TMARS associated with Bursch-Schneider and cemented DMC) and the other with a Ganz reinforcement device associated with a cemented DMC. We acknowledge than these two failures of cementless fixation for the DMC with early aseptic loosening could be considered as technical errors related to the implantation of a cementless DMC in a case of non-diagnosed pelvic discontinuity and a case associated with sclerotic acetabular bone that was not prone for cementless fixation (Fig. 3) (Table 3).

**Table 3:** Re-revision case data

Case	Sex	Age	BMI	Cause of revision	Type of revision	Paprosky classification^1^	Hauptfleisch MRI classification	Cause of re revision	Type of re revision	HHS preop	HHS 3 months	HHS 1 year	HHS final
1	M	51	23.7	ARMD	U	I/I	II	Instability: retroverted dual mobility cup	Change of cementless cup in well positioned	84.8	70.5	83.8	92
2	M	65	19.28	ARMD	C	I/I	I	Infection	Two-stage exchange	81	65.8	84.5	92.6
3	F	63	37.18	ARMD	U	I/IIa	I	Infection	Two-stage exchange	73.6	50.5	83.8	90.7
4	F	72	27.4	ARMD	U	I/IIa	III	Instability: adductor deficiency	Constrained liner in Ganz reinforcement cup	46.5	33	84.5	81.6
5	M	72	37.3	ARMD	C	I/IIB	II	Cup loosening	Ganz reinforcement device with a cemented DMC	73.5	45	83.8	80.5
6	M	92	24.91	ARMD	U	I/IIa	III	Cup loosening	Cup cage construct: TMARS associated with Bursch-Schneider and cemented DMC	65.6	34.5	82.6	92.6

*U* acetabular only revision, *C* complete

^1^Femoral/acetabular

In addition, periprosthetic femoral fractures occurred four and five years after two revision THA (3%) due a fall leading to femoral revision for a Vancouver B2 fracture and a re-operation for plate fixation for a Vancouver B1 fracture. No periprosthetic fracture of the acetabulum was observed.

### Surgical procedure

The mean operative time was 120 ± 104 min. The mean intraoperative blood loss was 600 ± 1200 ml. Intraoperative complications occurred during nine revision THA (13%). The most frequent intraoperative complications were related to insufficiency fractures related to ARMD-related osteolysis. There were five greater trochanteric fractures that were treated with a Dall-miles plate and cable fixation and three posterior acetabular wall fractures that were treated with plate fixation in addition to a Ganz reinforcement device.

## Discussion

Revision THA for failed large head MoM bearings is a challenging procedure potentially associated with high rates of complications and re-revisions, particularly in the context of ARMD [7, 20]. ARMD affects both the quality and quantity of bone and soft tissues surrounding the hip with abductor mechanism deficiency, which can potentially pose challenges for acetabular reconstruction during revision THA. Previous studies by Munro et al. [21] and Crawford et al. [5] reported high overall re-revision rates, up to 38%, following revision THA for ARMD. In our study, re-revision rate was 9%, while data from national registries describe an overall rate of re-revision ranging from 11 to 36% [22–24].

The main causes of re-revision were dislocation, aseptic loosening, and infection. However, to date, no study has specifically dedicated to the evaluate the outcomes of revision THA for failed large head MoM bearings with acetabular reconstruction using DMC [9, 11]. The most significant finding of this study was that DMC for revision of failed MoM bearings associated with ARMD effectively reduced the occurrence of dislocation and ensured a stable acetabular reconstruction, with low rates of aseptic loosening observed at the six year follow-up, [3, 21, 25]. However, even lower than the rates of up to 20% reported in previous study evaluating acetabular reconstruction with conventional constructs, dislocation remains a concern in our series with a rate of 8%, particularly in revision THA associated with severe soft tissue damage and hip abductor deficiency related to ARMD [26–28].

In our study, five patients (8%) experienced dislocation. Among them, two patients (3%) required re-revision due to recurrent instability for the reasons of abductor mechanism deficiency and malposition of the cup in retroversion. In literature, Borton et al. demonstrated dislocation rates of 11% and a re-revision rate of 33% when performed the acetabular reconstruction with conventional single mobility bearings such as ceramic-on-ceramic, ceramic-on-polyethylene, or metal-on-polyethylene bearings [25]. These findings are consistent with the results reported by Grammatopoulos et al. with dislocation rate of 18%, and Bonner et al. and Jennings et al. with dislocation rates of 15% and 22%, respectively [20, 29, 30]. The effectiveness of the dual mobility cup constructs was further emphasized by Klemt et al. and Colacchio et al., reporting no re-revisions and one revision for recurrent instability; respectively [28, 31]. However, it is worth noting that in cases of severe hip abductor deficiency, there may be limitations to the use of a dual mobility construct. In such instances, Rahman et al. utilized a constrained liner systematically to decrease the risk of dislocation, and their study demonstrated no instability during the follow-up period [27]. Despite a 3% re-revision rate for persistent instability, our findings support the advantages of the dual mobility cup construct over single mobility constructs in terms of instability prevention. However, in cases of severe hip abductor deficiency, the use of a constrained liner might be a preferred option.

Aseptic loosening of the cup is another common complication, particularly when using conventional coatings. In our study, two early aseptic loosening of the cup (3%) were reported with cementless dual mobility cup. They were related to a technical error related to an unrecognized acetabular bone defect. Munro et al. reported aseptic loosening rate of 12% by using cementless cup and non-osseointegration during the follow up, leading to re-revision. Similar findings were reported by Borton et al., who reported a 25% rate of aseptic loosening with cementless cup [24]. In contrast, acetabular component with highly porous coating, some authors have shown no re-revisions or very low rates (2.2%) when using ultraporous cups [5, 22]. In our study, no cases of aseptic loosening were observed when using a cemented cup in bony acetabulum or with the use of a supportive reinforcement ring. Furthermore, Matharu et al. and Liddle et al. have suggested the use of a reinforcement ring with a cemented cup to decrease the risk of aseptic loosening, especially in elderly patients or cases with severe bone defects [28, 29].

Prosthetic joint infection is also a significant concern following MoM revision for ARMD. Local inflammation and soft tissue damage and/or necrosis can increase the risk of prosthetic joint infection [26]. In our study, we observed early infections that resulted in re-revision or re-operation in three cases (4.6%). Our results were in accordance with periprosthetic infection rates ranging from 6 to 9% previously reported in literature [30–32].

Finally, ARMD causes increased bony fragility due to pseudotumor and bony cyst formation [5, 32]. Additionally, revision THA, regardless of the cause, is associated with an increased risk of fracture [33]. In our series, we observed intraoperative periprosthetic fragility fractures in 8 hips (12.5%), including femoral and acetabular posterior wall fractures, which required direct osteosynthesis and/or the use of a reinforcement ring.

Our study has several limitations that should be acknowledged. Firstly, this study was retrospective without control group, which limits our ability to compare the outcomes with alternative options for acetabular reconstruction, such as large heads or constrained acetabular components. Additionally, this study has a relatively mid-term follow-up period and includes a limited cohort of patients, although it should be noted that this cohort was continuous and represented the largest group specifically evaluating acetabular reconstruction with DMC in the context of revision THA for this specific indication. Moreover, all procedures were performed in a referral centre by a surgical team experienced in complex revision THA, which may affect the generalizability of the results to other settings with different patient populations and surgical expertise. Therefore, future studies with larger patient cohorts, longer follow-up periods, consideration of alternative components for acetabular reconstruction, and evaluation of various surgical teams' experiences are needed to provide a more comprehensive understanding of the outcomes in this specific context.

## Conclusion

Revision THA for failed large head MoM bearings is a complex procedure that can be associated with higher rates of complications and re-revisions, especially related to instability, aseptic loosening, and infection. This is particularly challenging when managing of ARMD that could result in significant bone loss and soft tissue damage. However, the use of dual mobility constructs (DMC) demonstrated effectiveness in addressing these challenges by providing a stable acetabular reconstruction with both cementless and cemented fixation options while reducing the risk of instability. It is important to note that dislocation remains a concern in these specific THA revisions, particularly in cases where there is hip abductor deficiency associated with ARMD. Alternative options such as constrained acetabular components should be carefully considered in those cases.

## Data Availability

All the data and material used for this study were saved in an anonymized file folder and available upon request.
